# Modifying Cellulose Acetate Mixed-Matrix Membranes for Improved Oil–Water Separation: Comparison between Sodium and Organo-Montmorillonite as Particle Additives

**DOI:** 10.3390/membranes11020080

**Published:** 2021-01-22

**Authors:** Micah Belle Marie Yap Ang, Kiara Pauline O. Devanadera, Alyssa Nicole R. Duena, Zheng-Yen Luo, Yu-Hsuan Chiao, Jeremiah C. Millare, Ruth R. Aquino, Shu-Hsien Huang, Kueir-Rarn Lee

**Affiliations:** 1 R&D Center for Membrane Technology, Department of Chemical Engineering, Chung Yuan Christian University, Taoyuan 32023, Taiwan; zhengyenluo@hotmail.com (Z.-Y.L.); ychiao@uark.edu (Y.-H.C.); 2 School of Chemical, Biological, and Materials Engineering and Sciences, Mapúa University, Manila 1002, Philippines; kpdevanadera@gmail.com (K.P.O.D.); alyduena@gmail.com (A.N.R.D.); jcmillare@mapua.edu.ph (J.C.M.); 3 Department of Chemical Engineering, University of Arkansas, Fayetteville, AR 72701, USA; 4 General Education Department, Colegio de Muntinlupa, Mayor J. Posadas Avenue, Sucat, Muntinlupa City 1770, Metro Manila, Philippines; ruthraquino@yahoo.com; 5 Department of Chemical and Materials Engineering, National Ilan University, Yilan 26047, Taiwan; 6 Research Center for Circular Economy, Chung Yuan Christian University, Taoyuan 32023, Taiwan

**Keywords:** montmorillonite, mixed-matrix membrane, ultrafiltration, oil–water separation, cellulose acetate

## Abstract

In this study, cellulose acetate (CA) mixed-matrix membranes were fabricated through the wet-phase inversion method. Two types of montmorillonite (MMT) nanoclay were embedded separately: sodium montmorillonite (Na-MMT) and organo-montmorillonite (O-MMT). Na-MMT was converted to O-MMT through ion exchange reaction using cationic surfactant (dialkyldimethyl ammonium chloride, DDAC). Attenuated total reflectance-Fourier transform infrared (ATR-FTIR) and X-ray photoelectron spectroscopy (XPS) compared the chemical structure and composition of the membranes. Embedding either Na-MMT and O-MMT did not change the crystallinity of the CA membrane, indicating that the nanoclays were dispersed in the CA matrix. Furthermore, nanoclays improved the membrane hydrophilicity. Compared with CA_Na-MMT_ membrane, CA_O-MMT_ membrane had a higher separation efficiency and antifouling property. At the optimum concentration of O-MMT in the CA matrix, the pure water flux reaches up to 524.63 ± 48.96 L∙m^−2^∙h^−1^∙bar^−1^ with over 95% rejection for different oil-in-water emulsion (diesel, hexane, dodecane, and food-oil). Furthermore, the modified membrane delivered an excellent antifouling property.

## 1. Introduction

Oily wastewater is one of the major contributors to water pollution globally. It is a type of wastewater mixed with oil with various range of concentrations and types of oil such as fats, hydrocarbons, food oil, diesel, gasoline, and kerosene. Large volumes of oily wastewater are produced from various industrial sources such as food processing, general metalworking, transportation, and oil and gas production [1,2,3]. The wastewater produced by the oil industry discharges not only oil and grease, but it also contains toxic components such as harmful chemicals and dissolved minerals, which can harm aquatic resources and human health [4,5,6]. Industrial oily wastewater can be categorized into three types: free-floating oil, which can be removed by mechanical gravity separation; unstable oil and water mixture that can be broken by mechanical and chemical separation; and stable oil and water emulsions that require sophisticated treatment [7].

Traditional methods for oil–water separation like gravity separation, skimming, and flotation, are only useful for free-floating oil mixtures with an oil droplet size of >150 μm and dispersed oil size range of 20–150 μm, but cannot be used to treat oil and water emulsion with <20 μm droplet size [8]. However, these methods have low efficiency and high operational costs. Combination of the following methods has been reported to treat the oily wastewater, including small droplet size: floatation, coagulation, biological treatment, and membrane separation technology [6].

Advanced membrane technology is a promising method to remove oily wastewater with small droplet size. Membrane separation has become the key process to treat oily wastewater [9]. The membrane serves as the semipermeable barrier between two phases that regulate the transportation of liquid between those two phases [10]. Certain types of membrane technology can be used for oil–water separation: microfiltration, ultrafiltration, nanofiltration, and reverse osmosis.

Ultrafiltration (UF) is the most suitable for the oil–water separation process due to its high efficiency in removing micron-sized oil droplets, ease in operation, and low energy consumption [9]. Polymeric materials have been used for the fabrication of UF membranes since it is inexpensive, and easy to fabricate and modify. Polymers such as polyethersulfone [4,11,12], polysulfone [9,13,14], polyvinylidene fluoride [15,16,17], and CA [18,19,20,21,22] are used as the matrix to fabricate UF membranes. CA is a biodegradable polymer that is usually used for aqueous-based separation, i.e., reverse osmosis and UF techniques [23]. Compared to other polymers, CA produced higher separation efficiency and has better affinity with water. Moreover, CA has an excellent chlorine resistance and is also inexpensive because it can be obtained from sustainable resources [24]. However, because of the intrinsic property of the CA membrane, it cannot meet the demand in terms of productivity and efficiency. Improving the hydrophilicity and antifouling property of CA membranes provides a way to prevent the deposition of the oil in the membrane surface and to improve the water transport through the membrane [25].

Several methods are proposed to improve the separation performance and longevity of the polymeric membranes for oil–water separation. These methods are surface modification [26,27], blending another hydrophilic polymer [28,29,30], and embedding nanoparticles in the polymeric matrix. Mixed-matrix membranes or polymer-inorganic membranes are fabricated by adding nanoparticle additives into the polymer matrix [31]. Nanoparticles such as graphene oxide [12,32,33,34,35], silica [22,36], titanium dioxide [37,38,39], nanoclay [40,41,42], halloysite nanotubes [43], nanowires [44], and silver nanoparticles [45] had been used to embed in the polymeric matrix. For example, Wan Ikshan et al. [43], synthesized halloysite nanotube-hydrous ferric dioxide through chemical precipitation. When they embedded it into polyethersulfone matrix, membrane hydrophilicity, and antifouling property was enhanced. Abdalla et al. [34] functionalized graphene oxide using aspartic acid. The aspartic functionalized graphene oxide was embedded into the polysulfone matrix. In their antifouling test, aspartic functionalized graphene oxide modified polysulfone membrane had higher flux recovery than that of their pristine membrane. Lai et al. [46] prepared a mixed matrix membrane of polyethersulfone containing dual-nanofiller. The dual-nanofillers used in their work were manganese oxide and titanium dioxide. At the optimal ratio of manganese oxide and titanium dioxide in the membrane, the modified membrane delivered higher separation efficiency and better antifouling property than pristine membrane. Pang et al. [47] also added two types of nanofiller in the polyethersulfone, multiwalled carbon nanotube and zinc oxide. Incorporating the multiwalled carbon nanotube improved the membrane porosity, whereas the zinc oxide enhances the antibacterial property of the membranes.

Nanoclay is a 2-D nanoparticle, which can be obtained naturally. It is abundant and easy to process. MMT is a type of nanoclay that is abundant in nature with hydrophilic oxides groups. It is a layered silicate composed of silica tetrahedral and alumina octahedral sheets. MMT can be incorporated into polymer nanocomposites because of its high surface area, unique two-dimensional nanostructures, and programmable layer response. It is easy to modify through the ion-exchange reaction with the cationic monomer or polymers. The organic modification of MMT can improve its capability of dispersing, gelling, adsorption, and nanocomposite in the organic systems [36]. Modified MMT enhances the mechanical properties, thermal properties, water permeability, porosity, and antifouling properties of a membrane.

Cellulose acetate membranes have been modified in a number of ways in previous studies. In this present study, different types of montmorillonite were embedded in them for the first time, with the main goal of conducting a comparative analysis of the contribution of the montmorillonite in effectively improving the composite membrane performance for oil–water separation. Moreover, the montmorillonite used was kind of unique, as it was obtained from a native mountain in the Philippines.

## 2. Materials and Methods

### 2.1. Materials

Philippine Na-MMT was supplied by Material Science Division, Industrial Technology Development Institute of the Department of Science and Technology, Taguig City, Philippines. Dialkyldimethyl ammonium chloride (DDAC), as modifier of Na-MMT, was a received from Hoechst Altiengesellschaft, Frankfurt, Federal Republic of Germany. CA 394-60 S powder was obtained from Arkema Colombes, France. N-Methyl-2-pyrrolidone (NMP), and dodecane were bought in Tedia High Purity, Fairfield, OH, USA. Sodium Dodecyl Sulfate (SDS) as surfactant of oil–water emulsion, was provided by Showa Chemical Industry Co. Ltd., Tokyo, Japan. Anhydrous ethanol and n-hexane were procured from Echo Chemical Co. Ltd., Miaoli, Taiwan. Food-grade oil was manufactured by Weiyi Enterprises Company Ltd., Kaohsiung, Taiwan. Diesel oil was purchased from CPC Corporation, Kaohsiung, Taiwan.

### 2.2. Synthesis of Organo-Montmorillonite

Organo montmorillonite was synthesized according to the work of Favre and Lagaly [48]. DDAC (=1.5 times of CEC of Na-MMT) was mixed with 100 g Na-MMT for 30 min in a blunger. The mixture was transferred to a stoppered glass container, where DDAC could react with Na-MMT for 72 h at 70 °C. The unreacted DDAC was removed through washing it several times using deionized water. Afterwards, it was dried at 80 °C for 24 h. The dried O-MMT was sieved using a 200-mesh. Na-MMT and O-MMT were stored in vacuum desiccator before used. The properties of Na-MMT and O-MMT can be found in our supporting information (Figures S1 and S2).

### 2.3. Preparation of Nanocomposite Membranes

The nanoclay was dispersed in NMP for 60 min using an ultrasonicator. After dispersing the nanoclay in NMP, it was moved to the magnetic mixer to stabilize the solution temperature at 80 °C and stirred at 80 rpm. The CA powder was added to the nanoclay/NMP solution with a total concentration of 15 wt % CA/NMP. Concentration of the Na-MMT and O-MMT was fixed at 0.2 wt % (based on the total amount of CA). The concentration of the preferred nanoclay was varied from 0–0.3 wt %. Controlled membrane was also prepared without adding nanoclay in the polymer solution. When the CA was completely dissolved in NMP, it was removed at the magnetic stirrer and degassed for 6 h at 30 °C.

The CA/nanoclay/NMP solution was cast (casting knife gap = 200 µm) on the glass-plate (A4-sized) covered with non-woven polyester. Afterwards, it was immediately immersed in water bath for phase-separation. Water was changed 4–5 times to completely removed the excess NMP. It was stored in distilled water before being used. CA_X_ represents the modified membranes, where X is Na-MMT or O-MMT.

### 2.4. Characterization of Montmorillonites and Membranes

The chemical analysis of the nanoclays and membranes was analyzed using an attenuated total reflectance-Fourier transform infrared (ATR-FTIR) spectroscopy (Perkin Elmer Spectrum 100 FTIR Spectrometer, Waltham, MA, USA) and X-ray photoelectron spectroscopy (XPS, VG Kalpha ThermoFisher Scientific, Inc., Waltham, MA USA). Field emission scanning electron microscopy (FESEM, S-4800, Hitachi Co., Tokyo, Japan) and atomic force microscopy (AFM, NanoScope^®^ V, Bruker, Billerica, MA, USA) were used to describe the membrane morphology and surface roughness. X-ray diffraction (XRD, Model D8 Advance Eco, Bruker, Billerica, MA, USA) determined the crystallinity of the MMTs and membranes. The wettability properties of the membranes were obtained using an interfacial tensiometer (PD-VP Model, Kyowa Interface Science Co. Ltd., Niiza-City, Saitama, Japan).

The bulk porosities of membranes (*ε*, %) were determined through gravimetric analysis [49,50]. Wet membranes were cut into a certain size. The weight of the wet membrane ($m_{w}$, g) was measured. Afterwards, the membranes were dried in vacuum for over 24 h. Then the weight of dry membrane (md) was recorded. The porosity of the membrane was calculated using the following equation:

(1) $$\varepsilon =\frac{m_{w}-m_{d}}{A\times l\times \rho }$$

where *A* (m) and ρ (kg∙m^−3^) were referred to the surface area and density of the membrane, respectively, and l (m) was the thickness of the membrane.

The mean pore radius (*r_m_*) was calculated using the Guerour–Elford–Ferry equation, as follows:

(2) $$r_{m}=\sqrt{\frac{\left(2.9-1.75\varepsilon \right)8\eta lQ}{\varepsilon A\Delta P}}$$

where *η* (kg∙m^−3^∙s^−1^) was the viscosity of the water, *Q* (m^3^∙s^−1^) was the permeate volumetric flowrate and Δ*P* (Pa) was the transmembrane pressure.

### 2.5. Evaluation of Membrane Performance

A crossflow filtration device was used to evaluate the filtration performance of each membrane. Four membranes were tested simultaneously. The membrane was placed in a steel cell with an effective membrane area (*A*) of 12.57 cm^2^. The flow rate was maintained to 0.6–0.7 L∙min^−1^. The membranes were prepressurized at 1.5 bar for 60 min. The pressure was lowered at 1 bar to determine the pure water flux (*J*, L∙m^−2^∙h^−1^). The pure water flux was calculated using the following equation:

(3) $$J=\frac{m}{\rho At}$$

where *m* was the mass of the permeate (g) at a certain time (*t*, s), and *ρ* was the density of the water (1 kg∙L^−1^).

After measuring the pure water flux of each membrane, the feed was changed into oil–water emulsion. The oils used were diesel, hexane, dodecane, and food oil. The weight ratio of oil in water was 1:99 with 0.09 g∙L^−1^ of SDS. The system was stabilized for 10 min before sampling. The feed and permeate concentration were determined using a total organic carbon (TOC) analyzer, Vario TOC select (Elementar, Langenselbold, Germany). The oil rejection was calculated using the following equation:

(4) $$R=\frac{C_{f}-C_{i}}{C_{f}}\times 100\%$$

where *C_f_* (ppm) was the concentration of oil in the feed and *C_i_* (ppm) was the concentration of the oil in permeate.

### 2.6. Evaluation of Antifouling Property

Antifouling property of the membrane was accessed similar to our previous work [51]. The membrane was placed in the crossflow filtration setup. Then it was prepressurized at 1.5 bar for 1 h. Sample permeates were taken every ten minutes for 3 times and measured as $J_{w1}$. The feed was change into an emulsion solution. The flux when the feed was an emulsion was recorded as $J_{o}$. After sampling for 5 times, every 10 min, the membrane underwent backwashing for 20 min. This cycle was repeated twice. The pure water flux was determined again after 2 cycles and recorded as $J_{w2}$.

For the evaluation of antifouling properties, flux recovery ratios (FRR) were calculated using the formula:

(5) $$FRR=\frac{J_{W2}}{J_{w1}}\times 100\%$$

The flux loss from irreversible fouling (*R_ir_*) and reversible fouling (*R_r_*) were obtained using the equations:

(6) $$R_{ir}=\left(\frac{J_{w1}-J_{w2}}{J_{w1}}\right)\times 100\%$$

(7) $$R_{r}=\left(\frac{J_{w2}-J_{o}}{J_{w1}}\right)\times 100\%$$

## 3. Results and Discussion

### 3.1. Characterization of the Membranes

#### 3.1.1. Membrane Chemical Property

**Figure 1:**
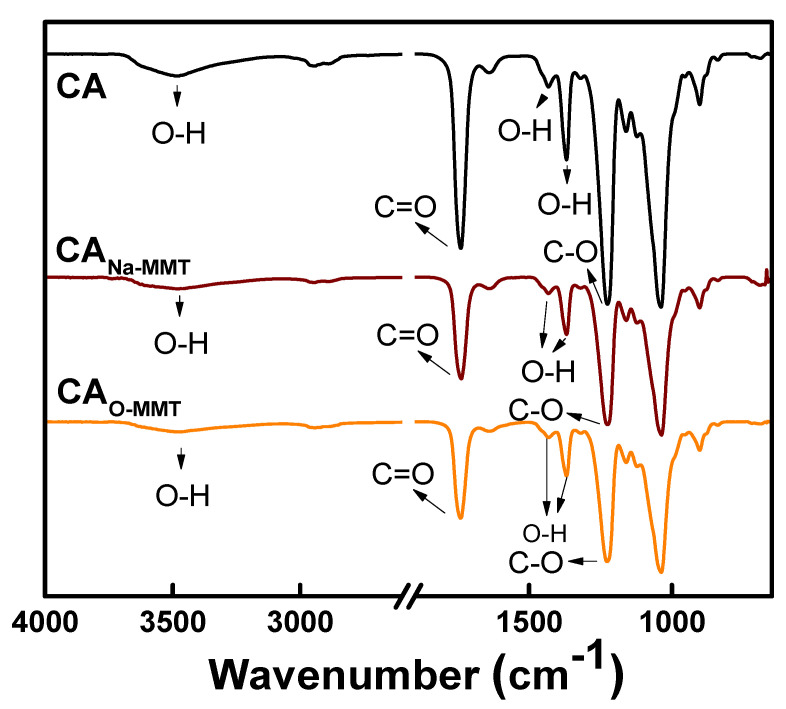
ATR-FTIR spectra of CA, CA_Na-MMT_, and CA_O-MMT_.

Figure 1 represents the ATR-FTIR spectra of the CA, CA_Na-MMT_, and CA_O-MMT_ membranes. The peak at 2941 cm^−1^ of all membranes was attributed to the aliphatic group (C-H). Peaks at 1743, 1440, and 1362 cm^−1^ corresponded to C=O, O-H, and C-O groups, respectively. The spectra of the MMTs (Figure S1) overlapped with the CA membranes, thus there were no significant changes on the CA membrane spectra after embedding MMTs. To further clarify the presence of the MMTS, XPS analysis (Table 1) validated the surface chemical composition. CA membrane only had the C and O element, whereas CA_Na-MMT_ and CA_O-MMT_ had Si, N, Na, Fe, Ti, K, Ca, P, Al, and Mg elements from MMT. CA_-Na-MMT_ (5.34%) had more elements that came from MMT than the CA_O-MMT_ (3.81%). Two possible reasons could attribute to this difference: (1) the O-MMT was well-dispersed in the CA matrix, thus less can be found on the surface; and (2) DDAC increased the d-spacing between MMT of O-MMT, making some MMT undetectable on the surface, because XPS only could detect up to 10 nm depth.

#### 3.1.2. Membrane Morphology and Structure

**Figure 2:**
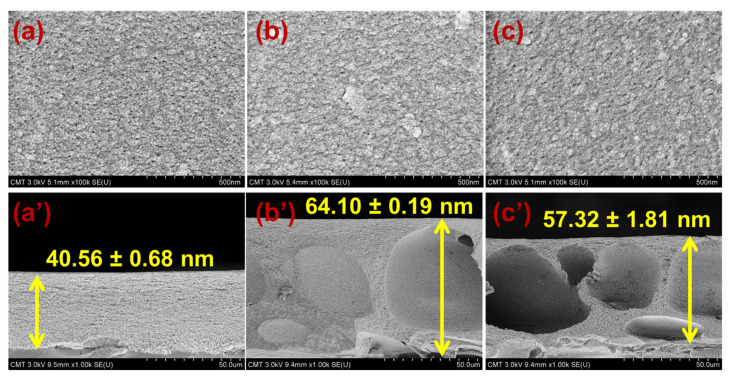
Surface and cross-sectional FESEM images of the membranes; (**a**,**a’**) CA; (**b**,**b’**) CA_NA-MMT_; and (**c**,**c’**) CA_O-MMT_.

**Figure 3:**
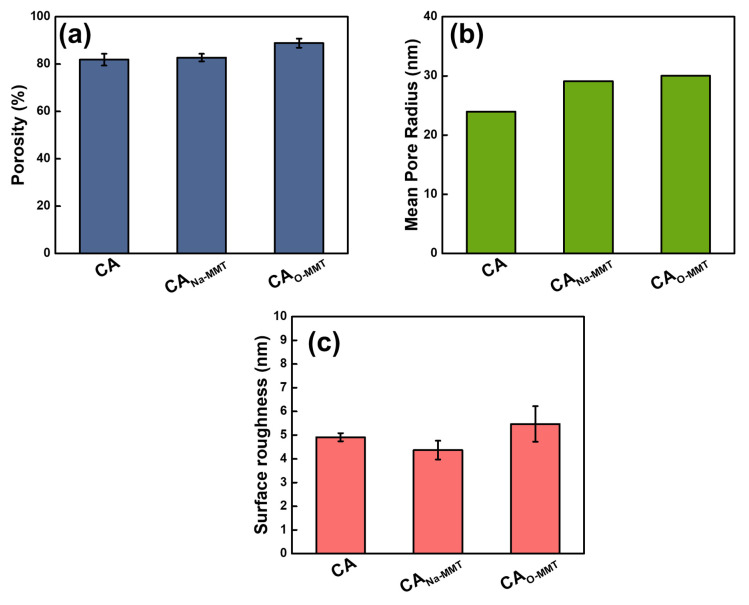
(**a**) Bulk porosity, (**b**) mean pore radius, and (**c**) surface roughness of the CA and modified membranes.

Figure 2 shows the FESEM images of CA, CA_Na-MMT_, and CA_O-MMT_. The pristine and modified CA membranes had a pore size of approximately 10 nm; however, because CA is prone to shrinkage when drying, this pore size is not their actual size. Therefore, the pore sizes were validated using the gravimetric method. To obtain the mean pore radius using flow–velocity filtration, the porosity of the membranes was also measured. Figure 3 indicates the porosity, mean pore radius, and surface roughness of pristine and modified membranes. CA_O-MMT_ (88.83%) was the most porous membrane. This is because O-MMT speed up the demixing rate of the CA membrane, leading to formation of the porous structure. Furthermore, CA_O-MMT_ had the largest mean pore radius (30.01 nm) and roughest surface (5.47 ± 0.75 nm). Rougher surface indicated that the membrane surface was more porous.

Figure 2a’–c’ present the cross-sectional FESEM images of the membranes. CA membrane had no macrovoids, which is similar to our previous work [51]. The lacy structure of pristine membranes was caused by the slow demixing rate of CA during phase separation. After embedding Na-MMT or O-MMT, macrovoids appeared on the modified CA membranes. The presence of macrovoids indicates that the solvent-nonsolvent exchange in modified membranes was faster than pristine CA membrane. Adding hydrophilic nanoparticles accelerated the demixing rate of the polymer solution with nonsolvent because hydrophilic nanoparticles had a strong affinity with water. Furthermore, adding MMTs made the polymer solution be thermodynamically unstable, resulting in a thicker membrane.

#### 3.1.3. XRD and Water Contact Angle

**Figure 4:**
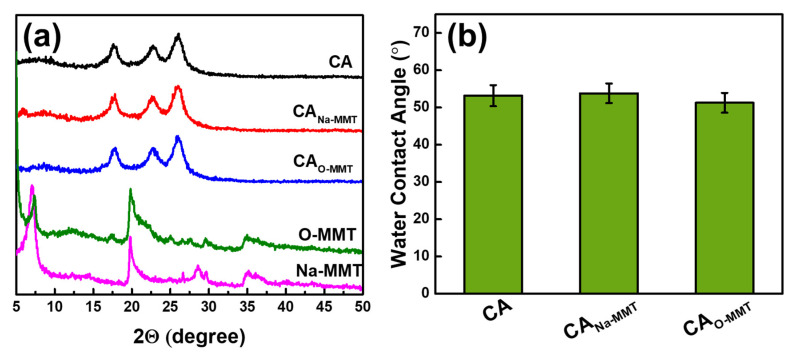
(**a**) XRD pattern of particles and membranes and (**b**) water contact angle of membranes.

Figure 4a illustrates the XRD pattern of the membranes and MMT. Compared with Na-MMT, O-MMT had a new peak at 5°, indicating an increase in d-spacing when DDAC modified the Na-MMT. When the nanoclay was embedded into the CA matrix, no change in XRD spectra was observed. This is because the concentration of the MMT in the polymeric matrix was low. Either using Na-MMT or O-MMT, both nanoclay could be well dispersed in the CA matrix. Figure 4b presents the water contact angle of the membranes. Compared with CA and CA_Na-MMT_, CA_O-MMT_ had the lowest contact angle of 51.27° ± 2.66°. The lower the contact angle, the more advantageous in an oil–water separation test.

### 3.2. Membrane Performance and Antifouling Property

**Figure 5:**
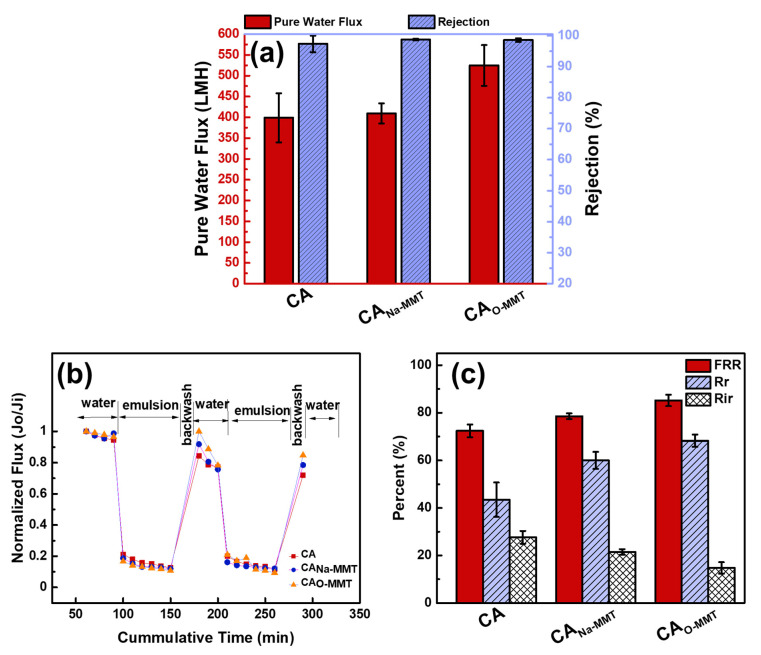
(**a**) Membrane performance and (**b**,**c**) antifouling property of CA, CA_Na-MMT_, and CA_O-MMT_.

Figure 5a describes the membrane performance of CA, CA_Na-MMT_, and CA_O-MMT_. CA membrane had a pure water flux of 398.74 ± 59.00 L∙m^−2^∙h^−1^. No significant changes in pure water flux when Na-MMT was embedded, this was probably because CA_Na-MMT_ had the thickest membrane. Although the membrane was more porous and contained macrovoids, its thickness compensated the permeation flux of the membranes. Thus, CA_NA-MMT_ had an almost similar pure water flux (409.18 ± 24.34 L∙m^−2^∙h^−1^) with the pristine CA membrane. When O-MMT was incorporated to the CA matrix, the pure water flux increased to 524.63 ± 48.96 L∙m^−2^∙h^−1^. Adding O-MMT in the CA matrix made the membrane more porous, thinner, and more hydrophilic than when adding Na-MMT, resulting in a higher pure water flux. Nonetheless, all membranes had a high rejection for diesel oil (>98%).

Figure 5b represents the antifouling test of the membranes for 290 min. From this test, data from Figure 5c was calculated. CA_O-MMT_ shows the highest flux recovery ratio 85.21%, whereas it also exhibited the highest reversible fouling of 68.28%. A membrane with high reversible fouling had good antifouling property since it could be easily removed with physical cleaning by backwashing the membrane with the use of water. Meanwhile, a low irreversible fouling percentage corresponds to internal fouling on membrane pores, which is difficult to clean or remove. Thus, CA_O-MMT_ membrane had the highest membrane performance, and in the following section, the concentration of O-MMT in CA was optimized.

### 3.3. Effect of O-MMT Concentration on Membrane Performance

**Figure 6:**
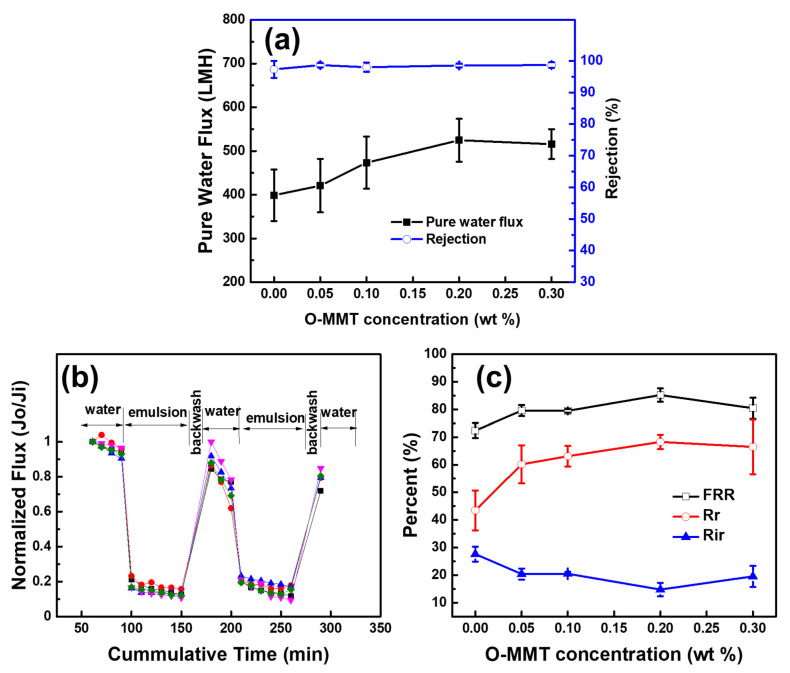
Effect of O-MMT concentration on (**a**) membrane performance and (**b**,**c**) antifouling property.

Figure 6a shows the effect of O-MMT concentration of membrane performance. Increasing the concentration of O-MMT from 0 to 0.2 wt % in the CA matrix, the pure water flux also increased from 398.74 ± 59.00 to 524.63 ± 48.96 L∙m^−2^∙h^−1^. However, more than 0.2 wt %, the pure water flux remained constant. At high concentration of O-MMT, O-MMT could also act as a barrier for water. Even if O-MMT could improve membrane hydrophilicity and porosity, when O-MMT acts as a barrier, this compensates the improvement in pure water flux, leading to no change in pure water flux [49]. Moreover, a high concentration of particle could also lead to aggregation of the nanoclays, leading to nonuniform distribution of the O-MMT in the membrane structure [52]. However, a different concentration of the O-MMT did not affect the oil separation using diesel (>98%). Figure 6b,c illustrates the effect of O-MMT concentration on the antifouling property of the CA membranes. The highest flux recovery ratio and reversible fouling were attained when the concentration of the O-MMT was 0.2 wt%. At high concentration of O-MMT, the nanoclay tended to agglomerate into the membrane structure, thus giving poor membrane performance. Therefore, 0.2 wt % O-MMT is the optimum concentration.

### 3.4. Membrane Performance Using Different Emulsion

**Figure 7:**
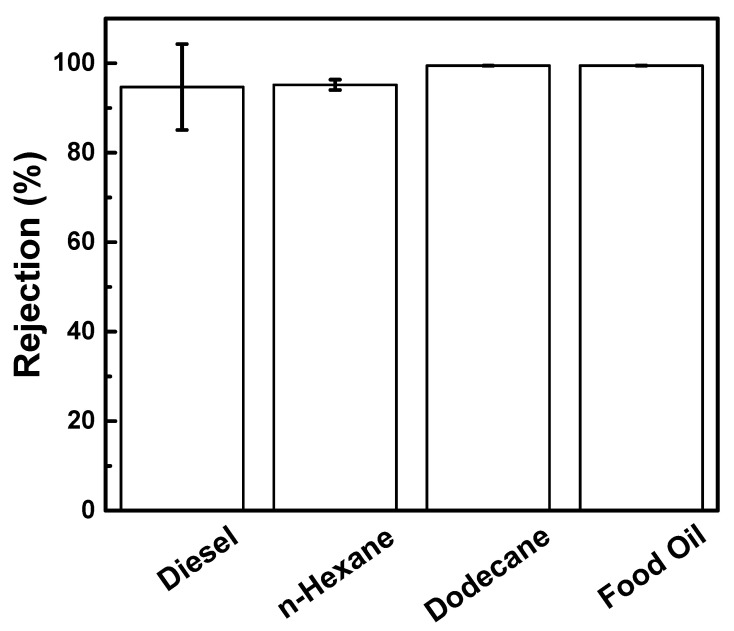
Rejection of CA_O-MMT_ membrane for different oil–water emulsion.

Different oil–water emulsion had different droplet sizes (Figure S3). Aside from diesel, hexane, dodecane, and food oil were also used (Figure 7). The CA_O-MMT_ membranes exhibited good separation performance for all the oil, where rejection was maintained more than 99% oil rejection. These results indicated that the O-MMT improved the membrane property at the optimum concentration of O-MMT. Furthermore, it can be used in different types of oil.

## 4. Conclusions

CA/nanoclay mixed-matrix membranes were fabricated through the wet-phase inversion method. O-MMT was obtained through modifying of Na-MMT by the ion-exchange reaction using DDAC. Incorporating nanoclay improved the demixing rate of the CA polymer solution, resulting in the formation of macrovoids. Compared with Na-MMT, embedding O-MMT into the CA matrix enhanced the membrane porosity, pore size, and hydrophilicity. No effect on membrane crystallinity when the nanoclays were embedded into the CA matrix. CA_O-MMT_ exhibited higher membrane performance and antifouling property than that of pristine CA and CA_Na-MMT_. The optimum concentration of O-MMT in CA was 0.2 wt % (based on the amount of CA). Furthermore, CA_O-MMT_ delivered high rate of rejections for different types of oil–water emulsion (diesel, n-hexane, dodecane, and food oil).

## Figures and Tables

**Table 1:** Surface elemental composition of the membranes using XPS analysis.

Element	CA	CA_Na-MMT_	CA_O-MMT_
C (%)	58.11	53	43.23
O (%)	41.89	41.66	52.94
Elements from Particle—Si, N, Na, Fe, Ti, K, Ca, P, Al, Mg (%)	-	5.34	3.81

## Supplementary Materials

The following are available online at https://www.mdpi.com/2077-0375/11/2/80/s1. Figure S1: ATR-FTIR spectra of nanoclays; Figure S2: Morphology of (a,a’) Na-MMT and (b,b’) O-MMT; Figure S3: Droplets size of different emulsion; Figure S4: Viscosity of polymer solutions.
