# Supplementary material for: Modifying Cellulose Acetate Mixed-Matrix Membranes for Improved Oil–Water Separation: Comparison between Sodium and Organo-Montmorillonite as Particle Additives
Source: Membranes (Basel). 2021 Jan 22;11(2):80. doi: 10.3390/membranes11020080 (PMC7911741; doi:10.3390/membranes11020080)
Supplement: Supplementary file 1 [file membranes-11-00080-s001.pdf]

Membranes

Electronic Supporting Information

Modifying Cellulose Acetate Mixed-Matrix Membranes for Improved Oil–Water Separation: Comparison between Sodium and Organo-Montmorillonite as Particle Additives

Micah Belle Marie Yap Ang [1,*], Kiara Pauline O. Devanadera [2], Alyssa Nicole R. Duena [2], Zheng-Yen Luo [1], Yu-Hsuan Chiao [1,3], Jeremiah C. Millare [2], Ruth R. Aquino [4], Shu-Hsien Huang [1,5,*] and Kueir-Rarn Lee [1,6,*]

[1] R&D Center for Membrane Technology and Department of Chemical Engineering, Chung Yuan Christian University, Taoyuan 32023, Taiwan; mbmyang@gmail.com (M.B.M.Y.A.); zhengyenluo@hotmail.com (Z.-Y.L.)

[2] School of Chemical, Biological, and Materials Engineering and Sciences, Mapúa University, Manila 1002, Philippines; kpdevanadera@gmail.com (K.P.D.); alyduena@gmail.com (A.N.R.D.); jcmillare@mapua.edu.ph (J.C.M.)

[3] Department of Chemical Engineering, University of Arkansas, Fayetteville, AR 72701, USA; ychiao@uark.edu (Y.-H.C.)

[4] General Education Department, Colegio de Muntinlupa, Mayor J. Posadas Avenue, Sucat, Muntinlupa City 1770, Metro Manila, Philippines; ruthraquino@yahoo.com

[5] Department of Chemical and Materials Engineering, National Ilan University, Yilan 26047, Taiwan; huangsh@niu.edu.tw (S.-H.H.)

[6] Research Center for Circular Economy, Chung Yuan Christian University, Taoyuan 32023, Taiwan

* Correspondence: mbmyang@gmail.com (M.B.M.Y.A.); huangsh@niu.edu.tw (S.-H.H.); krlee@cycu.edu.tw (K.-R.L.)

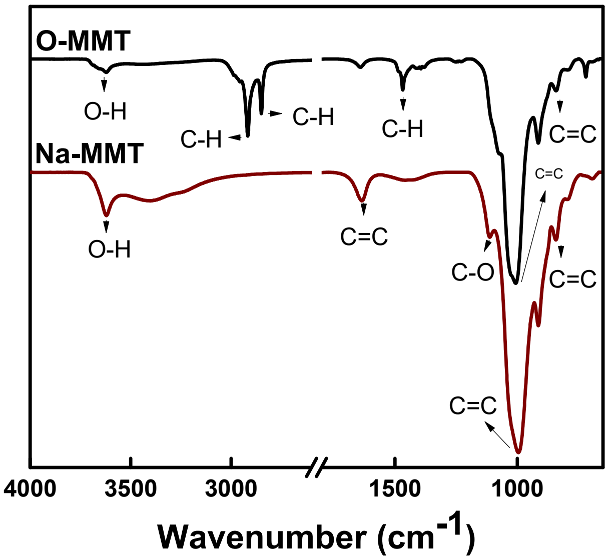

Figure S1. ATR-FTIR spectra of nanoclays.

Transmission electron microscopy (TEM, JEOL JEM-2100, Tokyo, Japan) was used to observe the morphology of the nanoclays. X-Ray Diffraction (XRD, Model D8 Advance Eco, Bruker, Billerica, MA, USA) determined the crystallinity of the MMTs and membranes.

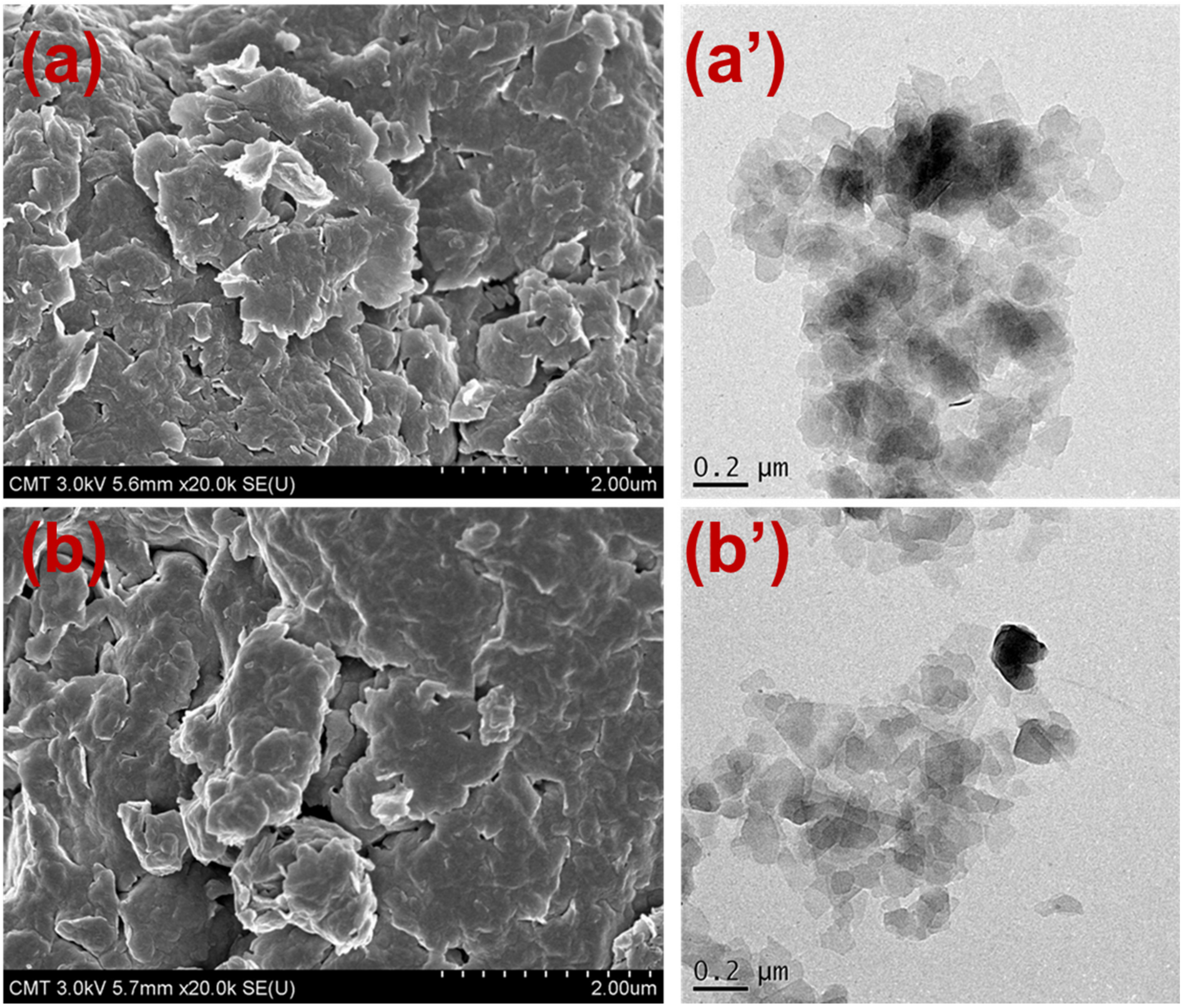

Figure S2. Morphology of (a,a') Na-MMT and (b,b') O-MMT.

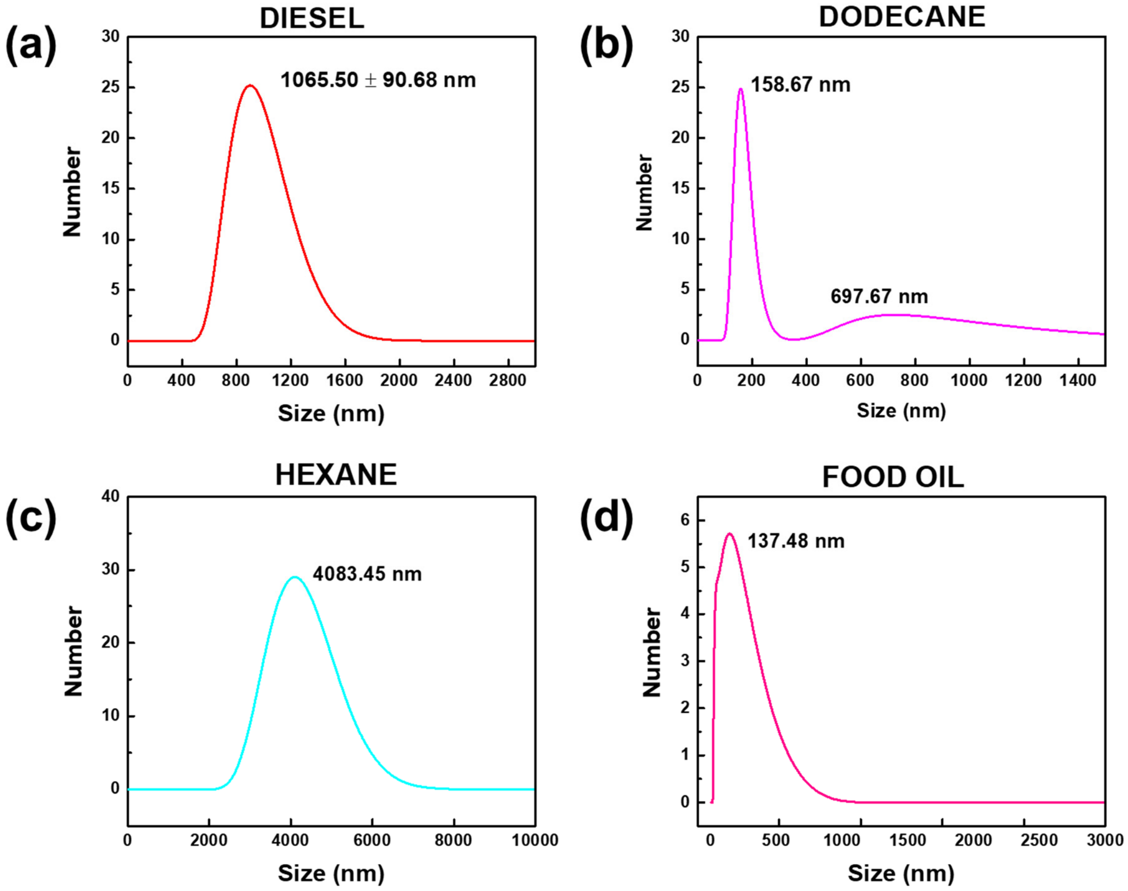

Figure S3. Droplets size of different emulsion.

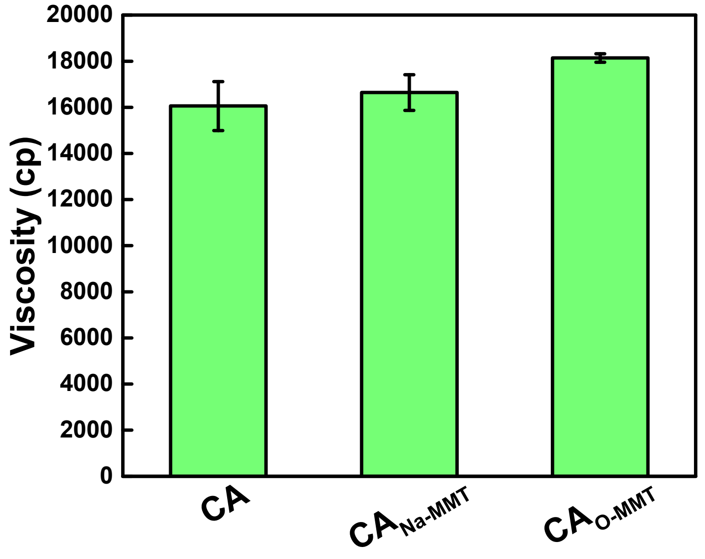

Figure S4. Viscosity of polymer solutions.
